# Predicting Contrast-Associated Acute Kidney Injury

**DOI:** 10.1001/jamanetworkopen.2025.0107

**Published:** 2025-03-05

**Authors:** Yunlin Feng, Min Jun, Amanda Y. Wang, Song Ren, Steven D. Weisbord, Rinaldo Bellomo, Marlies Ostermann, Clare Arnott, Martin Gallagher

**Affiliations:** 1 Department of Nephrology, Sichuan Provincial People’s Hospital, School of Medicine, University of Electronic Science and Technology of China, Sichuan Clinical Research Centre for Kidney Diseases, Chengdu, China; 2 The George Institute for Global Health, University of New South Wales, Sydney, New South Wales, Australia; 3 Concord Clinical School, University of Sydney, Sydney, New South Wales, Australia; 4 The Faculty of Medicine and Health Sciences, Macquarie University, Sydney, New South Wales, Australia; 5 Renal Section, Medicine Service, VA Pittsburgh Healthcare System, Pittsburgh, Pennsylvania; 6 Renal-Electrolyte Division, University of Pittsburgh School of Medicine, Pittsburgh, Pennsylvania; 7 Department of Critical Care, University of Melbourne, Melbourne, Victoria, Australia; 8 Intensive Care and Nephrology, King’s College London, London, United Kingdom; 9 Department of Critical Care, Guy’s & St Thomas’ Hospital, London, United Kingdom; 10 South West Sydney Clinical School, University of New South Wales, Sydney, New South Wales, Australia

## Abstract

This systematic review and meta-analysis updates a previous evaluation of the performance of risk-prediction models for contrast-associated acute kidney injury.

## Introduction

Contrast-associated acute kidney injury (CA-AKI), defined as AKI after exposure to a diagnostic or therapeutic radio-contrast agent,^1^ is an important clinical problem.^2^ CA-AKI has been reported as the third leading cause of AKI among inpatient populations and is associated with short- and long-term adverse outcomes.^3^ Prediction models for CA-AKI increased rapidly in number in recent years, especially in the cardiology setting, to help identify patients at high risk. We conducted this review to summarize the available evidence on risk-prediction models for CA-AKI.

## Methods

This systematic review and meta-analysis was performed using the PRISMA reporting guideline (eFigure in Supplement 1). As a meta-analysis, the research is exempt under 45 CFR §46.101(b)(4) from ethical review and informed consent. Critical appraisal was conducted using the PROBAST tool version 15/5/2019 (PROBAST Delphi group). Studies that developed a prediction model for CA-AKI that included at least 2 predictive variables and were published after the review by Silver et al^4^ were included. Discrimination data were pooled using summary receiver operating characteristic (sROC) curve analysis. Between-study heterogeneity was explored using subgroup analysis and meta-regression. Publication bias was assessed using funnel plot analysis. Methods details and extracted variables are presented in eTables 1 and 2 in Supplement 1. Statistical significance was set at a 2-tailed *P* < .05. Data analysis was performed using Stata version 14 MP (StataCorp) and R version 4.0.3 (R Project for Statistical Computing). Analyses were conducted between July 2023 and February 2024.

## Results

Overall, 64 studies with 64 prediction models for CA-AKI were included, with 9 models (14.1%) rated as having low risk of bias. The 5 most used predictive variables were baseline kidney function, past medical history, age, coronary artery disease, and cardiac function (Table).

**Table.** Predictive Variables in Included Prediction Models

Grouping variables	Total occurrences, No. (%)^a^	Predictive variables
Baseline kidney function	56 (14.1)	CKD, eGFR, CCR, serum creatinine, cystatin C, and serum BUN
Past medical history	50 (12.6)	Prior cardiac shock, prior cardiac arrest, prior MI, prior CVD, prior heart failure, diabetes, PVD, and stroke
Age	37 (9.3)	Age
CAD presentation	33 (8.3)	AMI, PCI status, ACS subtype, PCI indication, CAD presentation, multivessel PCI, emergency procedure, operation, and time to reperfusion
Cardiac function	22 (5.5)	NYHA class, LVEF, and Killip class
Blood pressure	20 (5.0)	SBP, hypotension, hypertension, and shock
Anemia	17 (4.3)	Anemia
CBC indices	15 (3.8)	SII, WBC count, neutrophil percentage, platelet count, RDW, NLR, lymphocyte count, and P-LCR
Contrast medium volume	15 (3.8)	Contrast medium volume
Cardiac biomarker	14 (3.5)	CK-MB, troponin I, myoglobin, and NT-pro-BNP
Liver function indices	12 (3.0)	Serum albumin, serum total bilirubin, LDH, and total protein
IABP	12 (3.0)	IABP
Inflammation biomarkers	12 (3.0)	Procalcitonin, Interleukin-18, and hsCRP
Blood lipid indices	11 (2.8)	Triglyceride, LDL-C, HDL-C, total cholesterol, and triglyceride-glucose index
Sex	8 (2.0)	Sex
Weight	7 (1.8)	Weight
Drug use	7 (1.8)	Loop diuretics use, β-blocker, antimicrobial drugs, and metformin use
Blood glucose	6 (1.5)	Blood glucose and HbA_1C_
Heart rate	5 (1.3)	Heart rate
Other	38 (9.6)	Height, uric acid, big ET-1, soluble klotho, KIM-1, osteopontin, CD5 antigen-like, free triiodothyronine, CHA_2_DS_2_-VASc score, AGEF score, Mehran score, serum sodium, serum calcium, serum potassium, fibrinogen-to-albumin ratio, fibrinogen, AT-III, INR, maximum AAA diameter, urinary system contrast blush grade, kidney PI, proteinuria, years since drinking, and admission source

Abbreviations: AAA, abdominal aortic artery; ACS, acute coronary syndrome; AGEF, age, estimated glomerular filtration rate and ejection fraction; AMI, acute myocardial infarction; AT-III, antithrombin III; BNP, brain natriuretic peptide; BUN, blood urea nitrogen; CAD, coronary artery disease; CBC, complete blood count; CCR, creatinine clearance rate; CHA_2_DS_2_-VASc, congestive heart failure, hypertension, age ≥75 years, diabetes, prior stroke/transient ischemic attack–vascular disease, age 65-74 years, sex category; CKD, chronic kidney disease; CK-MB, creatine kinase-MB; CVD, cardiovascular disease; eGFR, estimated glomerular filtration rate; ET-1, endothelin-1; HbA_1C_, hemoglobin A_1c_; HDL-C, high-density lipoprotein cholesterol; hsCRP, highly sensitive C reaction protein; IABP, intra-aortic balloon pump; INR, international normalized ratio; KIM-1, Kidney injury molecule-1; LDH, lactate dehydrogenase; LDL-C, low-density lipoprotein cholesterol; LVEF, left ventricular ejection fraction; MI, myocardial infarction; NLR, neutrophil-to-lymphocyte ratio; NT-pro-BNP, N-terminal pro–B-type natriuretic peptide; NYHA, New York Heart Association; PCI, percutaneous coronary intervention; PI, peaking intensity; P-LCR, platelet large cell ratio; PVD, peripheral vascular disease; RDW, red blood cell distribution width; SBP, systolic blood pressure; SII, systemic immune-inflammatory index; WBC, white blood cell.

^a^ Numbers in parentheses represent percentages of each combined variable in the total occurrence of predictive variables.

A total of 45 reviewed studies had requisite data to enable sROC curve analysis, resulting in a pooled *C* statistic of 0.83 (95% CI, 0.82-0.84) (Figure). The 95% confidence contour for the *C* statistic point estimate was smaller than that in Silver et al,^4^ whereas the 95% prediction contour was little changed. Neither subgroup analysis nor metaregression identified a discrete source of the heterogeneity observed. Asymmetry was observed based on visual inspection of funnel plots.

**Figure.**
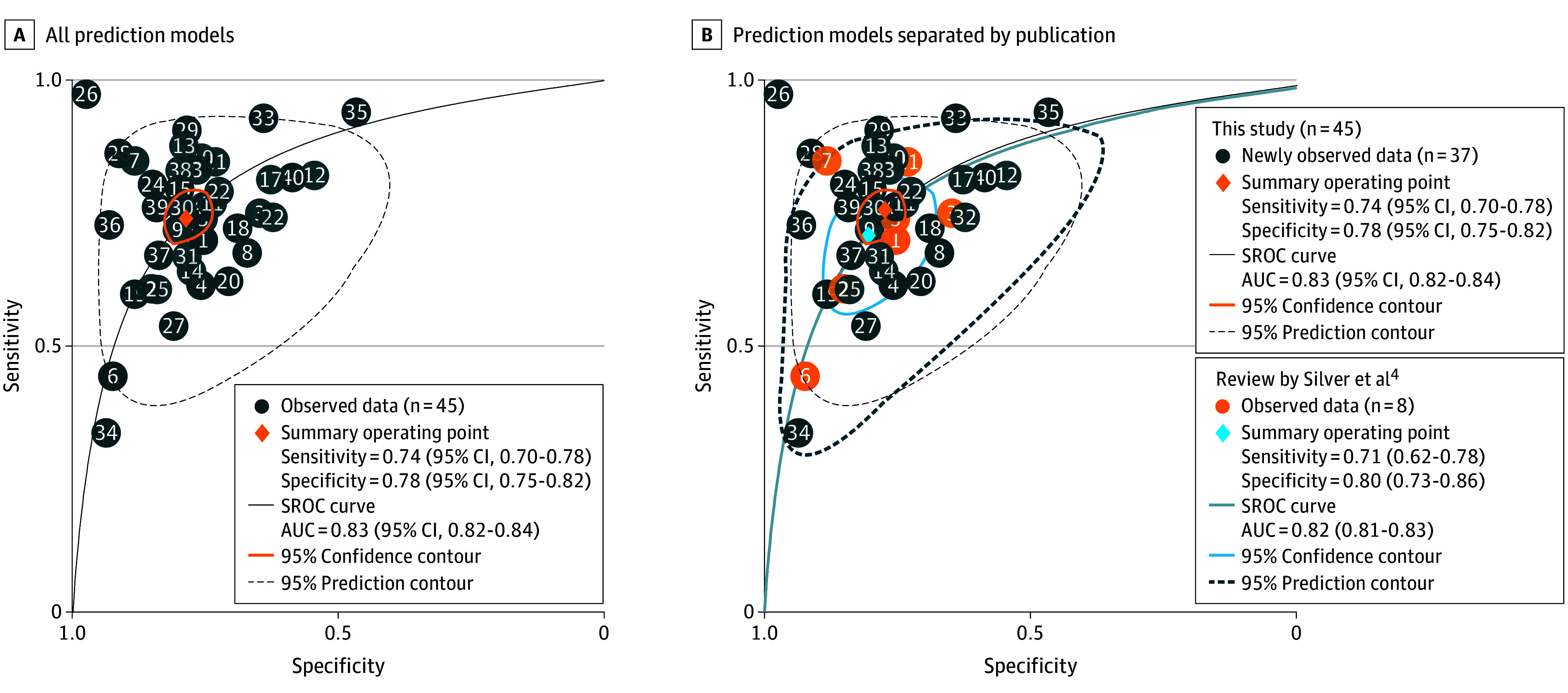
Summary Receiver Operating Characteristic (sROC) Curves of *C* Statistics A, The sROC curve of all 45 prediction models is presented. B, All sROC curves separated by prediction models included in the review by Silver et al^4^ review and those published afterward are presented. Among the 45 studies, 8 were included in the 2015 review by Silver et al^4^ and were separately summarized using the sROC curve analysis. AUC indicates area under the curve.

## Discussion

The number of CA-AKI prediction models has quadrupled since the 2015 review by Silver et al.^4^ This systematic review and meta-analysis found that despite a burgeoning literature and narrowing CIs around the point estimate of discrimination, substantial heterogeneity remained and there has been no meaningful improvement in the summary prediction estimate of model performance. Our approach of graphical presentation of sROC curves and reporting the breadth of the prediction interval better illustrates the heterogeneity and the resultant imprecision of any estimate of model performance.

It is important to bear in mind that the CI around the C statistic narrows as the total number of studies and participants increases given that it does not account for variations in study settings, patient characteristics, or methodologies. In contrast, the prediction interval does account for such variability in underlying studies and provides a more accurate representation of the imprecision in the estimate of model performance.^5,6^ Separating studies into 2 periods made it clear that while the CI for the *C* statistic was reduced with additional recent data, the prediction interval was not, suggesting that we are no closer to a recognized model for predicting CA-AKI that may have clinical or scientific utility.

Limitations include that predictive literature is dominated by retrospective studies, which are susceptible to selection, performance, and other biases. Performance bias is particularly challenging given that participants perceived to be at high risk of CA-AKI will often have the dose of contrast minimized or may be systematically excluded from datasets by never undergoing the procedure. A further challenge in interpreting this literature is the widespread use of modest changes in kidney function in the CA-AKI outcome definition, such as 25% increases in serum creatinine, which are more common but of dubious clinical significance.

## Notes

Supplement 1. **eTable 1.** Detailed methods of this study **eTable 2.** Basic characteristics of included prediction models **eFigure.** Flowchart of included studies
eReferences.

Supplement 2.
Data Sharing Statement
