# Supplementary material for: Predicting Contrast-Associated Acute Kidney Injury
Source: JAMA Netw Open. 2025 Mar 5;8(3):e250107. doi: 10.1001/jamanetworkopen.2025.0107 (PMC11883485; doi:10.1001/jamanetworkopen.2025.0107)
Supplement: Supplement 1. — eTable 1. Detailed methods of this study eTable 2. Basic characteristics of included prediction models eFigure. Flowchart of included studies eReferences. [file jamanetwopen-e250107-s001.pdf]

Supplemental Online Content

Feng Y, Jun M, Wang AY, et al. Predicting contrast-associated acute kidney injury: an updated systematic review and meta-analysis. *JAMA Netw Open*. 2025;8(3):e250107.
doi:10.1001/jamanetworkopen.2025.0107

eTable 1. Detailed methods of this study

eTable 2. Basic characteristics of included prediction models

eFigure. Flowchart of included studies

eReferences.

This supplemental material has been provided by the authors to give readers additional information about their work.

© 2025 Feng Y et al. *JAMA Network Open.*

Supplementary Table 1. Detailed Methods of this study.

Search Sources and Searches
A systematic review and meta-analysis was performed according to the Preferred Reporting Items for Systematic Reviews and Meta-Analyses (PRISMA) statement [1] by searching Embase via Ovid (from inception to May 23, 2023) and MEDLINE via PubMed (from inception to May 23, 2023) using medical subject headings and text words related to contrast-associated AKI and prediction models (sTable 1.1). The search was limited to publications in English. The protocol of this systematic review has been prospectively registered on PROSPERO (registration number: CRD42023428922).
Study Selection
Studies that developed a prediction model for CA-AKI that included at least two predictive variables were eligible for inclusion. A prediction model was defined as a statistical or machine-learning based model containing two or more independent predictive variables to estimate the occurrence of CA-AKI. It should be noted that this systematic review was based on a Silver et al's systematic review from 2015 [2]; therefore, publications in or before 2014 were excluded during the abstract and title screening. Reference lists from included articles were also manually screened to identify any other studies with potential to contribute data to the meta-analysis. Varying definitions of CA-AKI were used by individual studies, differing in the extent of increase in serum creatinine and/or time period after contrast exposure, but this was not used as a restriction for the included studies. Any study of a diagnostic or interventional procedure that had contrast medium was eligible for inclusion. Details of exclusion criteria are shown in sTable 1.2.<br>Of note, studies that examined only biomarkers as predictive variables or used a single ratio of two or more related variables to predict the occurrence of CA-AKI were excluded. For studies that had reported more than one model and clearly concluded on the best model, the best model was used. For studies that had equally reported more than one model, the model with the worst discrimination performance reflected by C-statistic was chosen.
Data Extraction
Two authors (FYL and RS) independently performed the literature search, screened the titles, abstracts, and full text articles, and extracted the data. Any disagreements during this process were resolved by consensus with participation of the senior author (MG).<br>Relevant data of prediction models from included studies were extracted using a standardized data extraction form based on the CHARMS (Critical Appraisal and Data Extraction for Systematic Reviews of Prediction Modelling Studies) checklist [3] and the TRIPOD (Transparent Reporting of a multivariable prediction model for Individual Prognosis Or Diagnosis) statement [4]. The extracted data included descriptions of study and model characteristics (see details in sTable 1.3).
Quality Assessment

Risk of bias of each prediction model was assessed using the PROBAST (Prediction model Risk Of Bias Assessment Tool) [5]. A model was rated as having low, high, or unclear overall risk of bias based on the assessment results of each domain in the PROBAST tool.

Outcome

The primary outcome in this meta-analysis was the occurrence of CA-AKI, which was recorded as per the definition in each included study. The details of the definitions and prediction window were also recorded.

Statistical analysis and data synthesis

Categorical variables were expressed as numbers and percentages. Continuous variables were expressed as mean and standard deviations or medians and interquartile interval as reported.

The discrimination data were pooled using the hierarchical summary receiver operating characteristics (HSROC) model with the results presented in a summary ROC (sROC) curve with a 95% confidence interval (CI) [6]. To generate the sROC curve, C-statistics and corresponding sensitivity and specificity data that were only reported graphically were extracted manually using GetData Graph Digitizer (version 2.26). Prediction models with insufficient data despite manual extraction were excluded from the sROC curve analysis.

Publication bias was evaluated using contour-enhanced funnel plot analysis [7] and the trim and fill method [8] to further explore the asymmetry in the funnel plot. Egger's regression test [9] was used to quantify the asymmetry in the funnel plot.

Data analysis was performed using Stata 14 MP (STATA, College Station, TX, USA) and R 4.0.3 (The R Core Team, R Foundation for Statistical Computing, Vienna, Austria) with R packages "forestplot", "metamisc", "metafor", "ggplot2", and "robvis".

© 2025 Feng Y et al. *JAMA Network Open.*

Supplementary Table 2. Basic characteristics of included prediction models.

Author	Region	Study Design	Study Period	Study Population	CA-AKI Definitions	Prediction Window	Study population				ROB
							Total No.	Age	Male (%)	CA-AKI No./%	
Tsai 2014[10]	USA	Retrospective	June 1, 2009 to June 30, 2011	Patients receiving PCI in NCDR CathPCI Registry	(AKIN) ≥0.3 mg/dL absolute or ≥1.5 folds relative increase in post-PCI creatinine	In hospital	947012	64.8±12.2	67.2	69453/7.3	Unclear
Bartholomew 2004[11]	USA	Retrospective	1993 to 2002	Patients receiving PCI in William Beaumont Hospital	≥1.0 mg/dl increase in serum Cr	≥8 hours after PCI	20479	NR	67.5	407/2.0	Unclear
Chen 2014[12]	China	Retrospective	January 2009 to May 2011	Patients receiving PCI at Tianjin Chest Hospital	an increase in serum Cr from pre-PCI levels to either ≥25% or by ≥0.5 mg/dL	within 5 days after PCI	2500	NR	69.3	418/16.7	High
Fu 2013[13]	China	Retrospective	January 2008 to December 2010	elderly (≥65y) patients undergoing PCI	a relative elevation ≥25% or an absolute increase of 0.5mg/dL in serum Cr, or a combination of both	48 to 72 hours after contrast exposure	945	NR	47.5	151/16.0	High
Gao 2014[14]	China	Retrospective	2005-2006, 2010	patients undergoing coronary angiography or PCI	an increase of serum Cr ≥44.2 μmol/l or ≥25% and beyond its upper limit of normal value	within 72 hours following the procedure	3945	NR	NR	177/4.5	High
Ghani 2009[15]	Kuwait	Prospective	March to May 2005	patients admitted for PCI in Kuwait chest disease hospital	an increase in serum Cr of ≥44.2 μmol/l	within 48 hours after the procedure	347	NR	NR	18/5.2	High
Gurm 2013[16]	USA	Retrospective	January 2010 to June 2012	patients undergoing PCI at 46 hospitals	≥0.5 mg/dl absolute increase in serum Cr level from baseline	1-7 days after the procedure	68573	65.0±12.2	66	1748/2.5	Unclear
Maioli 2010[17]	Italy	Retrospective	June 1, 2003 to December 31, 2004	patients undergoing elective coronary angiography or PCI	≥0.5 mg/dl absolute increase in serum Cr level	within 5 days after contrast exposure	1720	NR	NR	168/0.8	High
Marenzi 2004[18]	Italy	Prospective	January 1, 2001 to June 30, 2003	ST-segment elevation AMI patients undergoing primary PCI	an absolute increase in Cr >0.5 mg/dl after PCI	within 3 days after the procedure	208	62±11	79.3	40/19.2	High
Mehran 2004[19]	USA	Prospective	six years	patients undergoing PCI	increase ≥25% and/or ≥0.5mg/dl in serum Cr	within 48 hours after PCI	8357	NR	NR	NR	Unclear
Tziakas 2013[20]	Greece	Retrospective	September 2008 to January 2010	patients undergoing elective of emergency PCI	an increase of ≥25% or ≥0.5 mg/dl in pre-PCI serum Cr	within 48 hours after PCI	488	64±11	73.8	50/10.2	Unclear
Victor 2014[21]	India	Prospective	March 2008 to December 2011	patients undergoing PCI	an increase of ≥25% or ≥0.5 mg/dl in pre-PCI serum Cr	within 48 hours after PCI	1200	NR	NR	NR	High
Braet 2023[22]	USA	Retrospective	January 2013 to July 2021	patients for elective endovascular abdominal aortic aneurysm repair (EVAR) in the BMC2 database	rise in serum Cr from pre to post-procedure > 0.5mg/dl	during hospitalization	NR	NR	NR	NR	Unclear
Brown 2015[23]	The Netherland	Retrospective	January 01, 2009 to September 30, 2013	patients undergoing cardiac catheterization or PCI	increase of serum Cr ≥0.5 mg/dl	within 7 days after procedure	NR	NR	NR	NR	Unclear

© 2025 Feng Y et al. *JAMA Network Open*.

Buratti 2021[24]	Italy	Prospective	2004 to 2015	ST-segment elevation AMI patients undergoing primary PCI	absolute serum Cr increase ≥0.5 mg/dl	within 72 hours after PCI	NR	NR	NR	NR	High
Cetin 2023[25]	Turkey	Retrospective	March 2014 to June 2017	patients aged 18 years and older who underwent invasive coronary angiography with an iodine-based contrast media	serum Cr increase of ≥25% or ≥0.5 mg/dL compared with baseline	within 24-72 hours after CM exposure	NR	NR	NR	NR	High
Du 2021[26]	China	Retrospective	June 2017 to July 2000	patients undergoing PCI or CPB surgery	absolute increase in serum Cr ≥0.3 mg/dL	within 48 hours after operation	292	61±13	55.1	58/19.9	High
Duan 2017[27]	China	Prospective	2010 to 2012	patients receiving coronary angiography or PCI	increase in serum Cr by ≥50% or ≥0.3 mg/dL from baseline	within 48 hours after the procedure	1777	NR	NR	106/6.0	High
Efe 2021[28]	Turkey	Retrospective	NR	patients with angiography or PCI	increase in serum Cr by ≥25% or ≥0.5 mg/dL from baseline	within 72 hours after the procedure	486	61 (53-70)	60.1	78/16.0	High
Fujiwara 2022[29]	Japan	Retrospective	January 2010 to December 2019	patients undergoing elective or emergency PCI	increase of >0.5 mg/dl or >25% in the baseline serum Cr level	within 48-72 hours after CM exposure	1254	70.1±11.0	72.2	64/5.1	High
Gu 2019[30]	China	Prospective	2016	patients undergoing PCI	a 25% elevation in serum Cr or ≥0.5 mg/dL in serum Cr	within 48-72 hours after CM exposure	343	57.9±9.2	72	28/8.2	High
Guo 2017[31]	China	Retrospective	January 2015 to January 2017	patients undergoing coronary angiography or PCI	an absolute increase in serum Cr of ≥0.5 mg/dL or a relative serum Cr increase ≥25% from baseline	within 72 hours after CM exposure	245	65.7±11.0	68.2	34/13.9	High
Hu X 2017[32]	China	Prospective	May 2014 to July 2015	patients aged 18 years or older undergoing selected PCI	an increase in serum Cr of 0.5 mg/dL	within 48 to 72 hours after the procedure	192	54.8±12.2	54.2	32/16.7	High
Hu Y 2022[33]	China	Retrospective	August 2018 to December 2021	patients with ACS combined with diabetes who underwent PCI	serum Cr increased by ≥26.5μmol/L or at least 50% from baseline	within 7 days after CM exposure	1073		63.4	213/19.9	Unclear
Inohara 2015[34]	Japan	Retrospective	January 2011 to March 2013	consecutive PCIs registered in the Japanese Cardiovascular Database	an increase in serum Cr of 50% or 0.3 mg/dl compared with baseline	within 30 days after indexed procedure	5936	NR	NR	NR	High
Jeon 2019[35]	Korea	Retrospective	2009 to 2017	cancer patients undergoing contrast-enhanced CT	an increase of serum creatinine of 25%	within 2 to 6 days after contrast exposure	2240	NR	73.8	55/2.5	Unclear
Ji L 2015[36]	China	Retrospective	December 2011 to June 2013	patients who underwent PCI	an increase of creatinine >25% or > 44.2μmol from baseline	within 72 hours after CM exposure	805	NR	NR	NR	High
Koowattanatianchai 2019[37]	Thailand	Retrospective	June 1, 2017 to June 30, 2018	STEMI patients undergoing primary PCI	absolute increase in serum Cr ≥0.5 mg/dL or ≥ 25% from baseline	within 48 hours after primary PCI	217	NR	72.8	43/19.8	High
Lazaros 2016[38]	USA	Prospective	November 2006 to March 2009	patients who underwent emergency PCI for AMI within 12 hours from symptoms onset	increase in serum creatinine by ≥0.3 mg/dl within 48 hours, increase in serum creatinine to ≥1.5 times compared with baseline values within 72 hours, or urine volume <0.5 ml/kg/h for 6 hours	within 72 hours after PCI	348	NR	87.4	54/15.5	Unclear
Lei L 2020[39]	China	Prospective	January 2010 to October 2012	patients with CKD undergoing CAG or PCI	CI- AKI defined as serum Cr elevation ≥0.5 mg/dL or 25% from baseline	within the first 48–72 hours following contrast exposure	643	69.88±9.67	71.8	96/14.9	High
Li D 2022[40]	China	Retrospective	January 2009 to December 2019	patients undergoing CAG/PCI	an increase in serum Cr by ≥ 44 μmol/L or 25%	within 72 hours after contrast exposure	4295	70 (60, 70)	65.8	755/17.6	Low

© 2025 Feng Y et al. *JAMA Network Open*.

Li Y 2022[41]	China	Retrospective	January 13, 2015 to April 27, 2017	patients undergoing cardiac catheterization and PCI	increase of serum Cr ≥ 0.3 mg/dL or 1.5-fold	within 7 days	2560	NR	66.6	189/7.4	Unclear
Lian 2017[42]	China	Retrospective	August 2009 to February 2013	patients aged > 65 years who had undergone elective CAG	an increase in the serum Cr level by > 0.3 mg/dL or ≥ 50% from baseline	within 48-72 hours after CM exposure	1286	73.0 ± 4.9	67.7	83/6.5	High
Lin 2017[43]	China	Retrospective	January 2010 to December 2013	patients undergoing emergent PCI	an absolute increase in the serum Cr concentration by 0.5 mg/dl compared to the baseline	within 72 hours after CM exposure	692	62.1±12.4	80.9	55/7.9	Low
Liu L 2020[44]	China	Prospective	January 2010 to October 2012	patients aged ≥18 years with baseline hypoalbuminemia who were undergoing CAG or PCI	an increase of ≥0.3 mg/dL or 50% in serum Cr compared to baseline	within 48 to 72 hours after CAG/PCI	1272	NR	NR	119/9.4	High
Liu Y 2020[45]	China	Retrospective	January 2010 to December 2013	patients undergoing PCI/CAG	an increase in serum Cr ≥ 0.5 mg/dL from baseline	within 48–72 h after CAG or PCI	3469	NR	NR	115/3.3	Low
Liu Y 2015[46]	China	Prospective	January 2010 to September 2012	patients with CTO undergoing PCI	an absolute increase in serum Cr ≥0.5 mg/dL over baseline	within 48–72 h after CM exposure	629	62.5±11.0	83.3	17/2.7	High
Ma K 2022[47]	China	Retrospective	September 2018 to June 2021	non-ST-elevation ACS patients undergoing PCI	absolute increase of serum creatinine level ≥ 0.5mg/dl (44.2μmol/L) or ≥25% from pre-operative level	within 48–72 h after CM exposure	1156	NR	NR	NR	Unclear
Ma X 2023[48]	China	Retrospective	December 2017 to May 2020	patients with coronary artery disease undergoing elective PCI	a rise in serum creatinine levels ≥ 0.5mg/dL or 25% from baseline	within 72 hours after surgery	240	63 (54-68)	81.25	37/15.4	High
Mehran 2021[49]	USA	Retrospective	Jan 1, 2012 to Dec 31, 2020	patients undergoing PCI	an increase in serum creatinine by at least 50% or at least 0·3 mg/dL	within 48 h after PCI	20222	NR	NR	840/4.2	Low
Miura 2019[50]	Japan	Retrospective	March 2014 to March 2018	Patients who underwent transfemoral TAVI for severe aortic valve stenosis	an increase in serum Cr to 150–199%, an increase in serum Cr of ≥ 0.3 mg/dl	within 7 days after surgery	81	84.6±5.1	27.2	7/8.6	High
Mizuno 2015[51]	Japan	Retrospective	June 2011 to September 2013	STEMI patients undergoing primary PCI	a >25% increase or an absolute increase in serum creatinine of 0.5 mg/dl	within 3 days after PCI	102	NR	78.4	10/9.8	High
Mohebi 2022[52]	USA	Retrospective	2008 to 2011	patients undergoing coronary and/or peripheral angiography	an absolute increase of ≥0.3 mg/dL and/or ≥50% relative increase in serum creatinine after angiography compared with the preprocedure serum creatinine level	within 7 days after surgery	1114	NR	72	55/4.9	High
Ni 2019[53]	China	Retrospective	January 2010 and October 2012	patients undergoing CAG	an increase in serum Cr ≥0.5 mg/dL from baseline	within 72 hours after CAG	3469	62.9±11.1	76.7	115/3.3	High
Nusca 2021[54]	Italy	Retrospective	August 2016 through February 2018	patients undergoing PCI	an absolute increase in serum Cr ≥0.3 mg/dl	within 24 to 48 hours after contrast exposure	1344	69 (63, 76)	78	48/3.6	Low
Ozveren 2023[55]	Turkey	Retrospective	NR	patients with ST-elevation acute myocardial infarction who were treated with p-PCI	>25% or >0.5mg/dl increase of baseline creatinine values	within 48 hours after procedure	3057	58 (50, 67)	77.7	412/13.4	High
Qiu 2023[56]	China	Retrospective	January 2019 to June 2022	elderly (≥65 years) STEMI patients undergoing emergency PCI	an increase in serum Cr ≥26.5 μmol/L within 48 hours, or ≥1.5 times baseline serum Cr	within 7 days after surgery	542	NR	NR	74/13.7	High
Sun L 2020[57]	China	Retrospective	NR	Patients with AMI who underwent angiography therapy	an absolute increase in serum creatinine at 48 h of procedure by ≥ 0.3 mg/dl or an increase of more than or equal to 150% from its baseline value	within 7 days after procedure	1495	66.6±13.9	71.2	226/15.1	Low

© 2025 Feng Y et al. *JAMA Network Open*.

Sun Y 2020[58]	China	Retrospective	October 2018 and May 2019	patients who underwent PCI	a 0.5 mg/dl or 25% increase in serum creatinine levels	within 48 to 72 hours after CM exposure	394	64.4±11.1	61.4	48/12.1	High
Tang 2022[59]	China	Retrospective	December 2019 to December 2020	patients with T2DM who underwent CAG	An increase in serum Cr by ≥ 26.5 μmol/l (0.3 mg/dl) within 48 h or to ≥ 1.5times baseline within one week after administration of the contrast agent	within 7 days after contrast medium exposure	542	NR	56.6	51/9.4	High
Wybraniec 2017[60]	Poland	Prospective	2013 to 2015	patients with coronary artery disease subject to elective or urgent CA/PCI	≥50% relative or≥0.3 mg/dL absolute increase of serum creatinine concentration at 48 h post-procedurally	within 48 hours after procedure	95	65 (59, 71)	69.5	9/9.5	High
Yan 2023[61]	China	Retrospective	January 1, 2015 to December 31, 2020	patients with eGFR<60 and received CT or CT angiography with intravenous ICM	an increase in serum Cr by ≥0.3 mg/dl within 48 hours or an increase of 1.5 times of baseline serum Cr within 7 days after ICM administration	within 7 days after ICM administration	4218	65.1±13.5	64.1	440/10.4	Low
Yao 2021[62]	China	Retrospective	NR	patients with diabetes who underwent PCI with contrast exposure	an increase of either 25% or 0.5 mg/dL (44.2 μmol/L) in serum creatinine	within 72 hours after contrast exposure	1113	NR	NR	NR	High
Yin 2017[63]	China	Retrospective	September 2007 to January 2015	patients treated with CM for coronary angiography or percutaneous coronary intervention or received intravenous CM	an increase of 25% and/or 0.5 mg/dL in serum Cr within 72 hours above the baseline value	within 72 hours after contrast exposure	8800	55.3±14.8	62.1	1173/13.3	Unclear
Yuan 2022[64]	China	Retrospective	2013 to 2018	patients who underwent emergency PCI	an increase in serum Cr ≥0.5 mg/dL (44.2 μmol/L) above baseline	within 7 days after contrast medium exposure	3564	NR	NR	NR	Unclear
Zeng 2019[65]	China	Retrospective	January 2010 to December 2013	patients with DM undergoing CAG/PCI	an absolute increase in serum Cr by 0.5 mg/dL from the baseline	within 48-72 hours after contrast exposure	1157	64.46±10.5 5	72.3	45/3.9	Unclear
Zheng 2022[66]	China	Retrospective	1 January 2016 to 28 February 2019	patients who received gadolinium-based contrast agents	a 25% increase in the serum Cr value from baseline or as an increase of 0.5 mg/dL (44.2 μmol/L) in the absolute serum creatinine value	within 3 days of contrast agent administration	1404	64.8±13.6	56	55/3.9	High
Zhou 2018[67]	China	Retrospective	January 2013 to July 2017	Patients with AMI who underwent angiography therapy	an absolute increase of serum Cr of more than or equal to 0.3 mg/dL or increase to more than or equal to 150% from baseline	within 48-hour after CAG or PCI	920	NR	71.5	164/17.8	Low
Zhu 2023[68]	China	Retrospective	January 2019 to December 2021	patients with ACS and undergoing PCI	a 0.5 mg/dl (44.2 μmol/l) increase in serum Cr level from baseline or a 25% increase in relative value	within 48-72 hours after contrast exposure	1531	68.8±9.5	70.8	259/16.9	High
Huang 2018[69]	USA	Retrospective	June 1, 2009 to June 30, 2011	patients who underwent PCI procedures	A change in post-procedure creatinine larger than 0.3 mg/ dL or a 1.5-fold increase from before the procedure	NR	947091	64.8±12.2	67.2	69826/7.4	Unclear
Ando G. 2014[70]	USA	Retrospective	2009 to 2011	patients referred for primary PCI	absolute increase in serum creatinine concentration ≥0.5 mg/dL or an increase ≥25% from baseline within 72 hours after the administration of contrast medium, without any other plausible cause	within 72 hours	470	62±12	72	25/5.3	Unclear
Ibrahim 2019[71]	USA	Prospective	2008 to 2011	patients undergoing coronary and/or peripheral angiography with or without intervention	an abrupt reduction in kidney function with an absolute increase in serum Cr of more than or equal to 0.3 mg/dL, a percentage increase in sCr of ≥50%, or a	7 days	889	NR	71.8	43/4.8	Unclear

© 2025 Feng Y et al. *JAMA Network Open*.

					reduction in urine output within 7 days after contrast exposure						
Zambetti 2017[72]	USA	Retrospective	January 2008 to September 2013	patients referred for primary PCI treatment of STEMI	RIFLE criteria	Within 3-5 days	1050	58.2	67.5	136/13.0	Low
Ando G.[73]	Europe	Retrospective	January 2008 to June 2011	patients undergoing primary PCI for STEMI	an absolute increase in sCr ≥0.5 mg/dL or an increase≥25% from baseline	within 72 hours	481	62±12	18	25/5.2	High

Abbreviations: ACS, acute coronary syndrome; AMI, acute myocardial infarction; CA-AKI, contrast associated acute kidney injury; CAG, coronary angiography; CKD, chronic kidney disease; CM, contrast medium; Cr, creatinine; CTO, chronic total occlusion; No., number; NR, not reported; PCI, percutaneous coronary intervention; ROB, risk of bias.

© 2025 Feng Y et al. *JAMA Network Open*.

Supplementary Figure 1. PRISMA flow chart of the included studies.

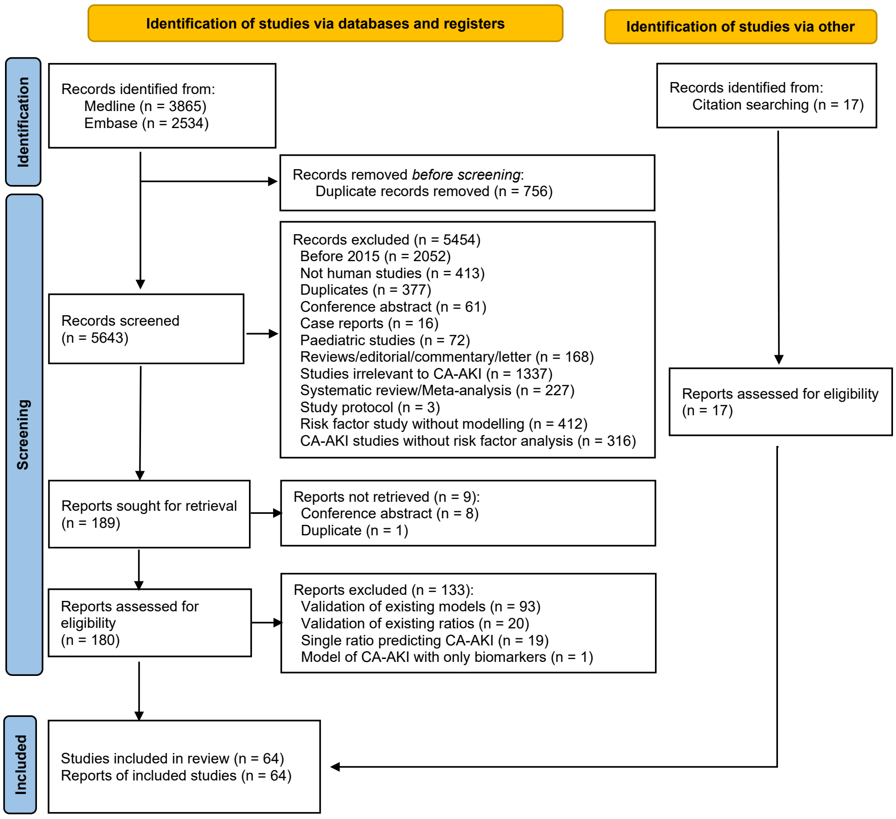

Abbreviations: CA-AKI, contrast-associated acute kidney injury.

© 2025 Feng Y et al. *JAMA Network Open*.

eReferences

1. Liberati A, Altman DG, Tetzlaff J, Mulrow C, Gotzsche PC, Ioannidis JP*, et al.* The PRISMA statement for reporting systematic reviews and meta-analyses of studies that evaluate healthcare interventions: explanation and elaboration. **BMJ (Clinical research ed) 2009**, 339**:** b2700.
2. Silver SA, Shah PM, Chertow GM, Harel S, Wald R, Harel Z. Risk prediction models for contrast induced nephropathy: systematic review. **BMJ 2015**, 351**:** h4395.
3. Moons KG, de Groot JA, Bouwmeester W, Vergouwe Y, Mallett S, Altman DG*, et al.* Critical appraisal and data extraction for systematic reviews of prediction modelling studies: the CHARMS checklist. **PLoS Med 2014**, 11(10)**:** e1001744.
4. Collins GS, Reitsma JB, Altman DG, Moons KG. Transparent reporting of a multivariable prediction model for individual prognosis or diagnosis (TRIPOD): the TRIPOD statement. **BMJ (Clinical research ed) 2015**, 350**:** g7594.
5. Wolff RF, Moons KGM, Riley RD, Whiting PF, Westwood M, Collins GS*, et al.* PROBAST: A Tool to Assess the Risk of Bias and Applicability of Prediction Model Studies. **Ann Intern Med 2019**, 170(1)**:** 51-58.
6. Higgins JP, Thompson SG. Quantifying heterogeneity in a meta-analysis. **Stat Med 2002**, 21(11)**:** 1539-1558.
7. Peters JL, Sutton AJ, Jones DR, Abrams KR, Rushton L. Contour-enhanced meta-analysis funnel plots help distinguish publication bias from other causes of asymmetry. **J Clin Epidemiol 2008**, 61(10)**:** 991-996.
8. Duval S, Tweedie R. Trim and fill: A simple funnel-plot-based method of testing and adjusting for publication bias in meta-analysis. **Biometrics 2000**, 56(2)**:** 455-463.
9. Egger M, Davey Smith G, Schneider M, Minder C. Bias in meta-analysis detected by a simple, graphical test. **BMJ 1997**, 315(7109)**:** 629-634.
10. Tsai TT, Patel UD, Chang TI, Kennedy KF, Masoudi FA, Matheny ME*, et al.* Validated contemporary risk model of acute kidney injury in patients undergoing percutaneous coronary interventions: insights from the National Cardiovascular Data Registry Cath-PCI Registry. **J Am Heart Assoc 2014**, 3(6)**:** e001380.
11. Bartholomew BA, Harjai KJ, Dukkipati S, Boura JA, Yerkey MW, Glazier S*, et al.* Impact of nephropathy after percutaneous coronary intervention and a method for risk stratification. **Am J Cardiol 2004**, 93(12)**:** 1515-1519.
12. Chen YL, Fu NK, Xu J, Yang SC, Li S, Liu YY*, et al.* A simple preprocedural score for risk of contrast-induced acute kidney injury after percutaneous coronary intervention. **Catheter Cardiovasc Interv 2014**, 83(1)**:** E8-16.
13. Fu N, Li X, Yang S, Chen Y, Li Q, Jin D*, et al.* Risk score for the prediction of contrast-induced nephropathy in elderly patients undergoing percutaneous coronary intervention. **Angiology 2013**, 64(3)**:** 188-194.
14. Gao YM, Li D, Cheng H, Chen YP. Derivation and validation of a risk score for contrast-induced nephropathy after cardiac catheterization in Chinese patients. **Clin Exp Nephrol 2014**, 18(6)**:** 892-898.
15. Ghani AA, Tohamy KY. Risk score for contrast induced nephropathy following percutaneous coronary intervention. **Saudi J Kidney Dis Transpl 2009**, 20(2)**:** 240-245.
16. Gurm HS, Seth M, Kooiman J, Share D. A novel tool for reliable and accurate prediction of

© 2025 Feng Y et al. *JAMA Network Open.*

renal complications in patients undergoing percutaneous coronary intervention. **J Am Coll Cardiol 2013**, 61(22)**:** 2242-2248.

17. Maioli M, Toso A, Gallopin M, Leoncini M, Tedeschi D, Micheletti C*, et al.* Preprocedural score for risk of contrast-induced nephropathy in elective coronary angiography and intervention. **J Cardiovasc Med (Hagerstown) 2010**, 11(6)**:** 444-449.
18. Marenzi G, Lauri G, Assanelli E, Campodonico J, De Metrio M, Marana I*, et al.* Contrast-induced nephropathy in patients undergoing primary angioplasty for acute myocardial infarction. **J Am Coll Cardiol 2004**, 44(9)**:** 1780-1785.
19. Mehran R, Aymong ED, Nikolsky E, Lasic Z, Iakovou I, Fahy M*, et al.* A simple risk score for prediction of contrast-induced nephropathy after percutaneous coronary intervention: development and initial validation. **J Am Coll Cardiol 2004**, 44(7)**:** 1393-1399.
20. Tziakas D, Chalikias G, Stakos D, Apostolakis S, Adina T, Kikas P*, et al.* Development of an easily applicable risk score model for contrast-induced nephropathy prediction after percutaneous coronary intervention: a novel approach tailored to current practice. **Int J Cardiol 2013**, 163(1)**:** 46-55.
21. Victor SM, Gnanaraj A, S V, Deshmukh R, Kandasamy M, Janakiraman E*, et al.* Risk scoring system to predict contrast induced nephropathy following percutaneous coronary intervention. **Indian Heart J 2014**, 66(5)**:** 517-524.
22. Braet DJ, Graham NJ, Albright J, Osborne NH, Henke PK. A Novel Preoperative Risk Assessment Tool to Identify Patients at Risk of Contrast-Associated Acute Kidney Injury After Endovascular Abdominal Aortic Aneurysm Repair. **Annals of Vascular Surgery 2023**.
23. Brown JR, MacKenzie TA, Maddox TM, Fly J, Tsai TT, Plomondon ME*, et al.* Acute Kidney Injury Risk Prediction in Patients Undergoing Coronary Angiography in a National Veterans Health Administration Cohort With External Validation. **J Am Heart Assoc 2015**, 4(12).
24. Buratti S, Crimi G, Somaschini A, Cornara S, Camporotondo R, Cosentino N*, et al.* A preprocedural risk score predicts acute kidney injury following primary percutaneous coronary intervention. **Catheter Cardiovasc Interv 2021**, 98(2)**:** 197-205.
25. Çetin M, Acehan F, Kundi H, Yakıcı IE, Katipoglu B, Duran G*, et al.* A novel risk prediction tool for contrast-induced nephropathy in patients with chronic kidney disease who underwent diagnostic coronary angiography. **European review for medical and pharmacological sciences 2023**, 27(8)**:** 3430-3437.
26. Du Y, Wang XZ, Wu WD, Shi HP, Yang XJ, Wu WJ*, et al.* Predicting the risk of acute kidney injury in patients after percutaneous coronary intervention (PCI) or cardiopulmonary bypass (CPB) surgery: Development and assessment of a nomogram prediction model. **Medical Science Monitor 2021**, 27**:** e929791.
27. Duan C, Cao Y, Liu Y, Zhou L, Ping K, Tan MT*, et al.* A New Preprocedure Risk Score for Predicting Contrast-Induced Acute Kidney Injury. **Can J Cardiol 2017**, 33(6)**:** 714-723.
28. Efe SC, Keskin M, Toprak E, Arslan K, Oz A, Guven S*, et al.* A Novel Risk Assessment Model Using Urinary System Contrast Blush Grading to Predict Contrast-Induced Acute Kidney Injury in Low-Risk Profile Patients. **Angiology 2021**, 72(6)**:** 524-532.
29. Fujiwara W, Ishii H, Sobue Y, Shimizu S, Ishiguro T, Yamada R*, et al.* A simple proteinuria-based risk score predicts contrast-associated acute kidney injury after percutaneous coronary intervention. **Sci Rep 2022**, 12(1)**:** 12331.
30. Gu G, Yuan X, Zhou Y, Liu D, Cui W. Elevated high-sensitivity C-reactive protein combined

with procalcitonin predicts high risk of contrast-induced nephropathy after percutaneous coronary intervention. **BMC Cardiovasc Disord 2019**, 19(1)**:** 152.

31. Guo BL, Ouyang FS, Yang SM, Liu ZW, Lin SJ, Meng W*, et al.* Development of a preprocedure nomogram for predicting contrast-induced acute kidney injury after coronary angiography or percutaneous coronary intervention. **Oncotarget 2017**, 8(43)**:** 75087-75093.
32. Hu X, Zhuang XD, Li Y, Li FF, Guo Y, Du ZM*, et al.* A Nomogram to Predict Contrast Induced Nephropathy in Patients Undergoing Percutaneous Coronary Intervention. **Int Heart J 2017**, 58(2)**:** 191-196.
33. Hu Y, Wang X, Xiao S, Sun N, Huan C, Wu H*, et al.* A Clinical Nomogram Based on the Triglyceride-Glucose Index to Predict Contrast-Induced Acute Kidney Injury after Percutaneous Intervention in Patients with Acute Coronary Syndrome with Diabetes Mellitus. **Cardiovascular therapeutics 2022**, 2022**:** 5443880.
34. Inohara T, Kohsaka S, Abe T, Miyata H, Numasawa Y, Ueda I*, et al.* Development and validation of a pre-percutaneous coronary intervention risk model of contrast-induced acute kidney injury with an integer scoring system. **Am J Cardiol 2015**, 115(12)**:** 1636-1642.
35. Jeon J, Kim S, Yoo H, Kim K, Kim Y, Park S*, et al.* Risk Prediction for Contrast-Induced Nephropathy in Cancer Patients Undergoing Computed Tomography under Preventive Measures. **Journal of Oncology 2019**, 2019**:** 8736163.
36. Ji L, Su X, Qin W, Mi X, Liu F, Tang X*, et al.* Novel risk score of contrast-induced nephropathy after percutaneous coronary intervention. **Nephrology (Carlton) 2015**, 20(8)**:** 544-551.
37. Koowattanatianchai S, Chantadansuwan T, Kaladee A, Phinyo P, Patumanond J. Practical risk stratification score for prediction of contrast-induced nephropathy after primary percutaneous coronary intervention in patients with acute ST-segment elevation myocardial infarction. **Cardiology Research 2019**, 10(6)**:** 350-357.
38. Lazaros G, Zografos T, Oikonomou E, Siasos G, Georgiopoulos G, Vavuranakis M*, et al.* Usefulness of C-Reactive Protein as a Predictor of Contrast-Induced Nephropathy After Percutaneous Coronary Interventions in Patients With Acute Myocardial Infarction and Presentation of a New Risk Score (Athens CIN Score). **Am J Cardiol 2016**, 118(9)**:** 1329-1333.
39. Lei L, Xue Y, Guo Z, Liu B, He Y, Liu J*, et al.* Nomogram for contrast-induced acute kidney injury in patients with chronic kidney disease undergoing coronary angiography in China: a cohort study. **BMJ Open 2020**, 10(5)**:** e037256.
40. Li D, Jiang H, Yang X, Lin M, Gao M, Chen Z*, et al.* An Online Pre-procedural Nomogram for the Prediction of Contrast-Associated Acute Kidney Injury in Patients Undergoing Coronary Angiography. **Frontiers in Medicine 2022**, 9**:** 839856.
41. Li Y, Chan TM, Feng J, Tao L, Jiang J, Zheng B*, et al.* A pattern-discovery-based outcome predictive tool integrated with clinical data repository: design and a case study on contrast related acute kidney injury. **BMC Med Inform Decis Mak 2022**, 22(1)**:** 103.
42. Lian D, Liu Y, Liu YH, Li HL, Duan CY, Yu DQ. Pre-Procedural Risk Score of Contrast-Induced Nephropathy in Elderly Patients Undergoing Elective Coronary Angiography. **Int Heart J 2017**, 58(2)**:** 197-204.
43. Lin KY, Zheng WP, Bei WJ, Chen SQ, Islam SM, Liu Y*, et al.* A novel risk score model for prediction of contrast-induced nephropathy after emergent percutaneous coronary intervention. **Int J Cardiol 2017**, 230**:** 402-412.
44. Liu L, Liu J, Lei L, Wang B, Sun G, Guo Z*, et al.* A prediction model of contrast-associated

© 2025 Feng Y et al. *JAMA Network Open.*

acute kidney injury in patients with hypoalbuminemia undergoing coronary angiography. **BMC Cardiovasc Disord 2020**, 20(1)**:** 399.

45. Liu Y, Chen S, Ye J, Xian Y, Wang X, Xuan J*, et al.* Random forest for prediction of contrast-induced nephropathy following coronary angiography. **Int J Cardiovasc Imaging 2020**, 36(6)**:** 983-991.

46. Liu Y, Liu YH, Tan N, Chen JY, Zhou YL, Duan CY*, et al.* Novel risk scoring for pre-procedural prediction of contrast-induced nephropathy and poor long-term outcomes among patients with chronic total occlusion undergoing percutaneous coronary intervention. **European Heart Journal, Supplement 2015**, 17(Supplement_C)**:** C34-C41.

47. Ma K, Li J, Shen G, Zheng D, Xuan Y, Lu Y*, et al.* Development and Validation of a Risk Nomogram Model for Predicting Contrast-Induced Acute Kidney Injury in Patients with Non-ST-Elevation Acute Coronary Syndrome Undergoing Primary Percutaneous Coronary Intervention. **Clinical interventions in aging 2022**, 17**:** 65-77.

48. Ma X, Mo C, Li Y, Chen X, Gui C. Prediction of the development of contrast-induced nephropathy following percutaneous coronary artery intervention by machine learning. **Acta Cardiologica 2023**.

49. Mehran R, Owen R, Chiarito M, Baber U, Sartori S, Cao D*, et al.* A contemporary simple risk score for prediction of contrast-associated acute kidney injury after percutaneous coronary intervention: derivation and validation from an observational registry. **Lancet 2021**, 398(10315)**:** 1974-1983.

50. Miura D, Yamada Y, Kusaba S, Nogami E, Yunoki J, Sakamoto Y*, et al.* Influence of preoperative serum creatinine level and intraoperative volume of contrast medium on the risk of acute kidney injury after transfemoral transcatheter aortic valve implantation: a retrospective observational study. **BMC research notes 2019**, 12(1)**:** 484.

51. Mizuno A, Ohde S, Nishizaki Y, Komatsu Y, Niwa K. Additional value of the red blood cell distribution width to the Mehran risk score for predicting contrast-induced acute kidney injury in patients with ST-elevation acute myocardial infarction. **Journal of cardiology 2015**, 66(1)**:** 41-45.

52. Mohebi R, van Kimmenade R, McCarthy C, Gaggin H, Mehran R, Dangas G*, et al.* A Biomarker-Enhanced Model for Prediction of Acute Kidney Injury and Cardiovascular Risk Following Angiographic Procedures: CASABLANCA AKI Prediction Substudy. **J Am Heart Assoc 2022**, 11(10)**:** e025729.

53. Ni Z, Liang Y, Xie N, Liu J, Sun G, Chen S*, et al.* Simple pre-procedure risk stratification tool for contrast-induced nephropathy. **Journal of Thoracic Disease 2019**, 11(4)**:** 1597-1610.

54. Nusca A, Mangiacapra F, Sticchi A, Polizzi G, D'Acunto G, Ricottini E*, et al.* Usefulness of Adding Pre-procedural Glycemia to the Mehran Score to Enhance Its Ability to Predict Contrast-induced Kidney Injury in Patients Undergoing Percutaneous Coronary Intervention Development and Validation of a Predictive Model. **Am J Cardiol 2021**, 155**:** 16-22.

55. Ozveren O, Tanalp AC, Tanboga IH, Karagoz A, Saygi M, Birdal O*, et al.* A new marker for the prediction of contrast induced-acute kidney injury following primary percutaneous coronary intervention: logarithm of haemoglobin-albumin product. **Acta Cardiologica 2023**.

56. Qiu H, Zhu Y, Shen G, Wang Z, Li W. A Predictive Model for Contrast-Induced Acute Kidney Injury After Percutaneous Coronary Intervention in Elderly Patients with ST-Segment Elevation Myocardial Infarction. **Clinical interventions in aging 2023**, 18**:** 453-465.

© 2025 Feng Y et al. *JAMA Network Open.*

57. Sun L, Zhu W, Chen X, Jiang J, Ji Y, Liu N*, et al.* Machine Learning to Predict Contrast-Induced Acute Kidney Injury in Patients With Acute Myocardial Infarction. **Frontiers in Medicine 2020**, 7**:** 592007.
58. Sun Y, Zheng D, Zhang Q, Li W. Predictive value of combining the level of fibrinogen and antithrombin iii for contrast-induced nephropathy in coronary artery disease patients undergoing percutaneous coronary intervention. **Biomedical Reports 2020**, 13(4)**:** 1-7.
59. Tang H, Chen H, Li Z, Xu S, Yan G, Tang C*, et al.* Association between uric acid level and contrast-induced acute kidney injury in patients with type 2 diabetes mellitus after coronary angiography: a retrospective cohort study. **BMC Nephrol 2022**, 23(1)**:** 399.
60. Wybraniec MT, Chudek J, Bozentowicz-Wikarek M, Mizia-Stec K. Prediction of contrast-induced acute kidney injury by early post-procedural analysis of urinary biomarkers and intra-renal Doppler flow indices in patients undergoing coronary angiography. **Journal of Interventional Cardiology 2017**, 30(5)**:** 465-472.
61. Yan P, Duan SB, Luo XQ, Zhang NY, Deng YH. Development and validation of a deep neural network-based model to predict acute kidney injury following intravenous administration of iodinated contrast media in hospitalized patients with chronic kidney disease: a multicohort analysis. **Nephrol Dial Transplant 2023**, 38(2)**:** 352-361.
62. Yao ZF, Shen H, Tang MN, Yan Y, Ge JB. A novel risk assessment model of contrast-induced nephropathy after percutaneous coronary intervention in patients with diabetes. **Basic Clin Pharmacol Toxicol 2021**, 128(2)**:** 305-314.
63. Yin WJ, Yi YH, Guan XF, Zhou LY, Wang JL, Li DY*, et al.* Preprocedural Prediction Model for Contrast-Induced Nephropathy Patients. **J Am Heart Assoc 2017**, 6(2).
64. Yuan Y, Qiu H, Hu X, Zhang J, Wu Y, Qiao S*, et al.* A risk score model of contrast-induced acute kidney injury in patients with emergency percutaneous coronary interventions. **Frontiers in Cardiovascular Medicine 2022**, 9**:** 989243.
65. Zeng JF, Chen SQ, Ye JF, Chen Y, Lei L, Liu XQ*, et al.* A simple risk score model for predicting contrast-induced nephropathy after coronary angiography in patients with diabetes. **Clin Exp Nephrol 2019**, 23(7)**:** 969-981.
66. Zheng H, Wang G, Cao Q, Ren W, Xu L, Bu S. A risk prediction model for contrast-induced nephropathy associated with gadolinium-based contrast agents. **Ren Fail 2022**, 44(1)**:** 741-747.
67. Zhou X, Sun Z, Zhuang Y, Jiang J, Liu N, Zang X*, et al.* Development and Validation of Nomogram to Predict Acute Kidney Injury in Patients with Acute Myocardial Infarction Treated Invasively. **Sci Rep 2018**, 8(1)**:** 9769.
68. Zhu Y, Qiu H, Wang Z, Shen G, Li W. Predictive value of systemic immune-inflammatory index combined with CHA2DS2-VASC score for contrast-induced acute kidney injury in patients with acute coronary syndrome undergoing percutaneous coronary intervention. **International Urology and Nephrology 2023**.
69. Huang C, Murugiah K, Mahajan S, Li SX, Dhruva SS, Haimovich JS*, et al.* Enhancing the prediction of acute kidney injury risk after percutaneous coronary intervention using machine learning techniques: A retrospective cohort study. **PLoS Med 2018**, 15(11)**:** e1002703.
70. Ando G, de Gregorio C, Morabito G, Trio O, Saporito F, Oreto G. Renal function-adjusted contrast volume redefines the baseline estimation of contrast-induced acute kidney injury risk in patients undergoing primary percutaneous coronary intervention. **Circ Cardiovasc Interv 2014**, 7(4)**:** 465-472.

© 2025 Feng Y et al. *JAMA Network Open.*

71. Ibrahim NE, McCarthy CP, Shrestha S, Gaggin HK, Mukai R, Magaret CA*, et al.* A clinical, proteomics, and artificial intelligence-driven model to predict acute kidney injury in patients undergoing coronary angiography. **Clin Cardiol 2019**, 42(2)**:** 292-298.
72. Zambetti BR, Thomas F, Hwang I, Brown AC, Chumpia M, Ellis RT*, et al.* A web-based tool to predict acute kidney injury in patients with ST-elevation myocardial infarction: Development, internal validation and comparison. **PLoS One 2017**, 12(7)**:** e0181658.
73. Ando G, Morabito G, de Gregorio C, Trio O, Saporito F, Oreto G. Age, glomerular filtration rate, ejection fraction, and the AGEF score predict contrast-induced nephropathy in patients with acute myocardial infarction undergoing primary percutaneous coronary intervention. **Catheter Cardiovasc Interv 2013**, 82(6)**:** 878-885.

© 2025 Feng Y et al. *JAMA Network Open.*
