# Supplementary material for: Predicting Contrast-Associated Acute Kidney Injury
Source: JAMA Netw Open. 2025 Mar 5;8(3):e250107. doi: 10.1001/jamanetworkopen.2025.0107 (PMC11883485; doi:10.1001/jamanetworkopen.2025.0107)
Supplement: Supplement 2. — Data Sharing Statement [file jamanetwopen-e250107-s002.pdf]

Data Sharing Statement

Feng. Predicting Contrast-Associated Acute Kidney Injury: An Updated Systematic Review and Meta-Analysis. *JAMA Netw Open*. Published online March 5, 2025. doi:10.1001/jamanetworkopen.2025.0107

Data
Data available: Yes
Data types: Data (not involving human participants)
How to access data: The data generated and analyzed during this study is available from the correspondence on reasonable request.
When available: With publication

Supporting Documents
Document types: None

Additional Information
Who can access the data: The data generated and analyzed during this study is available from the correspondence on reasonable request.
Types of analyses: The data generated and analyzed during this study is available from the correspondence on reasonable request.
Mechanisms of data availability: The data generated and analyzed during this study is available from the correspondence on reasonable request.
